# Identification of prognostic and bone metastatic alternative splicing signatures in bladder cancer

**DOI:** 10.1080/21655979.2021.1964252

**Published:** 2021-08-17

**Authors:** Runzhi Huang, Zixuan Zheng, Shuyuan Xian, Jiayao Zhang, Jingyi Jia, Dianwen Song, Penghui Yan, Huabin Yin, Peng Hu, Xiaolong Zhu, Zongqiang Huang, Tong Meng, Jie Zhang

**Affiliations:** a Key Laboratory Of Spine And Spinal Cord Injury Repair And Regeneration Tongji University, Ministry Of Education, Shanghai, China; b Division Of Spine, Department Of Orthopedics, Tongji Hospital Affiliated To Tongji University School Of Medicine, Shanghai, China; c Tongji University School Of Medicine (Shanghai Pulmonary Hospital), Shanghai, China; d School Of Mathematical Sciences Of Tongji University, Shanghai, China; e Department Of Orthopedics, Shanghai General Hospital, School Of Medicine, Shanghai Jiaotong University, Shanghai, China; f Department Of Orthopedics, The First Affiliated Hospital of Zhengzhou University, Zhengzhou, China; g Tongji University Cancer Center, Shanghai Tenth People’s Hospital, Tongji University School Of Medicine, Shanghai, China

**Keywords:** Alternative splicing, bladder cancer, prognosis, bone metastasis, signaling pathway

## Abstract

Bladder cancer (BLCA), originating from the epithelium of the urinary bladder, was the second most common malignancy in the urinary system with a high metastasis rate and poor post-metastasis prognosis. Alternative splicing events (ASEs) were regarded as important markers of tumor progression and prognosis, however, their roles in bladder cancer bone metastasis have not been recognized. In this study, we constructed a predictive model based on ASEs and explored the molecular mechanism of ASEs in BLCA bone metastasis, based on data from the Cancer Genome Atlas (TCGA) and TCGASpliceSeq databases. We proposed the hypothesis that the splicing events of ITGB4 was regulated by the splicing factor JUP, and this regulation might play a key role in BLCA bone metastasis through the glycosphingolipid biosynthesis ganglio series pathway.

## Introduction

Bladder cancer (BLCA), originating from the epithelium of the urinary bladder, is the fourth most common cancer in men and the second most common malignancy in the urinary system [1]. In the past few years, the incidence and mortality of bladder cancer have been rising gradually [2]. In 2018, there were 549,000 new cases of bladder cancer and 200,000 deaths worldwide [3]. There are two subtypes of bladder cancer, non-muscle invasive bladder cancer (NMIBC) and muscle invasive bladder cancer (MIBC). A large number of patients occurred bone metastasis at the terminal stage. By then, a total tumor resection is difficult. Even with cisplatin chemotherapy, bladder cancer patients with bone metastasis could not survive more than 14–15 months [4]. Thus, there is a pressing need to explore the mechanism of bone metastasis and predict the prognosis of patients with bladder cancer.

Alternative splicing (AS) is a pivotal determinant of genome complexity and an important mechanism for generating proteome diversity [5]. In the human genome, about 95% of the genes are alternatively spliced [6,7], in turn, it also couples with the complexity of the genome. Thus, AS takes part in diverse mRNA isoforms spliced and protein variants translated. In this process, splicing factor (SF) works as regulatory catalyst of alternative splicing events (ASEs). The aberrant AS of some genes and somatic mutations of SFs were frequently found in tumors. They might influence the protein–protein interactions in cancer-related pathways and modulate malignant transformation of cells, tumor cells invasion and metastasis [8]. Thus, identifying the dysregulated network of SFs and ASEs may provide the novel molecular biomarkers for prognosis, metastasis and therapy [9–12]. However, a comprehensive analysis of the prognostic value of ASEs in bladder cancer especially for bladder cancer bone metastasis is still lacking which aroused our interest.

In this study, RNA sequencing data and clinical information of BLCA patients were retrieved from TCGA database and ASE data were obtained from TCGASpliceSeq database [13]. Firstly, overall survival (OS) associated ASEs (OS-SEs) were identified and OS related pathways were subsequently figured out based on top 20 OS-ASEs of each splicing pattern. Then, a prognostic model was constructed using lasso regression model for character selection and using multivariate Cox regression analysis for calculation of risk score. Finally, a co-expression network was constructed based on SFs, OS-SEs and KEGG pathways. A hypothesis was proposed that ASE of ITGB4 was regulated by the SF JUP, which plays an important role in BLCA bone metastasis through the glycosphingolipid biosynthesis ganglio series pathway. To overcome the shortage of in-silicon analysis, we attached multi-level validation of our hypothesis by searching different external databases and analyzing external datasets.

## Methods

### Data extraction

The study was approved by the Ethics Committee of the First Affiliated Hospital of Zhengzhou University (No. KEYAN-2018-LW-040). The bladder cancer RNA-seq data was downloaded from the cancer genome atlas (TCGA, https://tcga-data.nci.nih.gov/tcga/). The dataset contained 412 samples of primary BLCAs, including 23 samples with bone metastasis and 389 samples without bone metastasis. Matched alternative splicing events (ASEs) information of BLCA samples were downloaded from the TCGASpliceSeq database [13]. The ASE ID was consisted of the gene name, the ID number of the TCGASliceSeq database (AS ID) and alternative splicing type, for example, in the annotation term ‘STXBP2-47,123-AP’, the STXBP2 was the gene name, 47,123 was the AS ID and alternate promoter (AP) was the splicing pattern. Collection of clinic-pathological data including gender, age, TNM staging, clinical stage, grade, survival status, and survival time were also exported. Moreover, differential expression analysis was performed based on RNA-seq data from TCGA and Integrative Clinical Genomics of Metastatic Cancer known as MET500 [14]. Additionally, the single-cell RNA sequencing (scRNA-seq) data from Gene Expression Omnibus (GEO) (GSE164041, https://www.ncbi.nlm.nih.gov/geo/query/acc.cgi?acc=GSE164041) and the Assay for Transposase Accessible Chromatin with high-throughput sequencing (ATAC-Seq) data from the TCGA database (https://tcga-data.nci.nih.gov/tcga/) were exported to validate our hypothesis.

### Identification of OS-SEs and function enrichment analysis

Univariate Cox regression analysis was applied to identify splicing events associated with overall survival. The top 20 enriched terms of Gene Ontology (GO) term and KEGG pathway of the genes in OS-SEs were taken into a further analysis.

### Analysis of the prognostic values of the risk scores

The top 20 OS-SEs of each splicing pattern were picked for lasso regression model [15]. After removing OS-SEs that might cause over fitting, the risks of BLCA were calculated using multivariate Cox regression. Cross-validation based on the single dataset was utilized for model construction and the risk scores of each patient were calculated by the following formula:

risk score = βOS-SE1 × PSIOS-SE1 + βOS-SE2 × PSIOS-SE2 + • •••• + βOS-SEn × PSIOS-SEn

To discriminate and evaluate the efficiency of our model, the receiver operating characteristic curve (ROC) was generated, and the area under curve (AUC) was also calculated. Meanwhile, Kaplan–Meier survival analysis was also performed based on risk scores in our model. Moreover, the univariate and multivariate Cox regression model were implemented based on risk score, to estimate whether the risk score was an independent prognostic factor of BLCA prognosis.

### Cross-validation

For cross-validation, the total dataset was divided into training and testing datasets by a ratio of 3:7, based on lasso regression which was used to balance the proportion of dead people in each dataset. The model construction process, including lasso regression, multivariate Cox regression, K-M survival analysis and model validation was performed in training dataset, testing dataset and total dataset.

### Construction of the regulatory network of ASE and SF in BLCA

A total of 390 SFs were acquired from the SpliceAid2 database (http://www.introni.it/splicing.html) [16]. Co-expression analysis was implemented to identify regulation between SFs and OS-SEs. Regulation pairs with a correlation coefficient > 0.45 and p value < 0.001 were finally selected into the network.

### Identification of OS-SEs related KEGG pathways in BLCA bone metastasis

Gene Set Variation Analysis (GSVA) was executed to identify potential OS related pathways which maybe the downstream mechanism of OS-SEs. Then, univariate Cox regression analysis was performed to figure out OS related pathways among these potential mechanisms. We also identified bone metastasis associated and OS-SEs co-expressed pathways by non-parametric test and co-expression analysis. To clearly understand the molecular regulatory mechanism, co-expression analysis based on SFs and OS-related pathways and a co-expression network was finally generated.

### External database validation

To minimize bias, multiple databases including UALCAN [17], LinkedOmics [18], SurvExpress [19], Cancer Cell Line Encyclopedia (CCLE) [20], the human protein atlas (HPA) [21] and Gene Expression Profiling Interactive Analysis (GEPIA) [22] were applied to detect the expression of genes on the tissue and cellular levels.

### Validation based on scRNA-seq data

The scRNA-seq data of bladder carcinoma cells were exported from GEO (https://www.ncbi.nlm.nih.gov/geo/query/acc.cgi?acc=GSE164041) to validate the association and distribution of key genes in our hypothesis in BLCA. The R package ‘Seurat’ was utilized to read [23]. Genes expressed in less than 200 single cells or with a transcript counts more than 100,000 or less than 1500 were removed. After data normalization using the LogNormalize function, variable genes were identified using the ‘vst’ method. Principal component analysis (PCA) was performed to filter genes with high-impact based on variable genes. Then, top 20 PCs were further analyzed using the UMAP (Uniform Manifold Approximation and Pro-jection) method to figure out cellular clusters. Genes with the FDR < 0.5 and the absolute value of log2(FC) > 0.5 were defined as differently expressed. Cell annotation was finished using the singleR method [24] and CellMarker database [25].

### Validation based on ATAC- seq data

Accessible-chromatin was regarded as potential targeted site of transcription factor, enhancer, silencer and other regulatory elements. The chromatin-accessibility of our key factors were detected based on ATAC-seq data of 700 BLCA patients downloaded from the TCGA database.

### Statistical analysis

All statistical analyses were performed using R version 3.5.1 software (Institute for Statistics and Mathematics, Vienna, Austria; www.r-project.org) (Package: edgeR [26], ggplot2 [27], glmnet [28], preprocessCore [29], survminer and timeROC. For all statistical analyses, only two-sided P < 0.05 was examined statistically significant. UpSetplots were applied to visualize the associations between genes and the different types of SEs.

## Results

### Analysis of ASEs in BLCA

**Figure 1.**
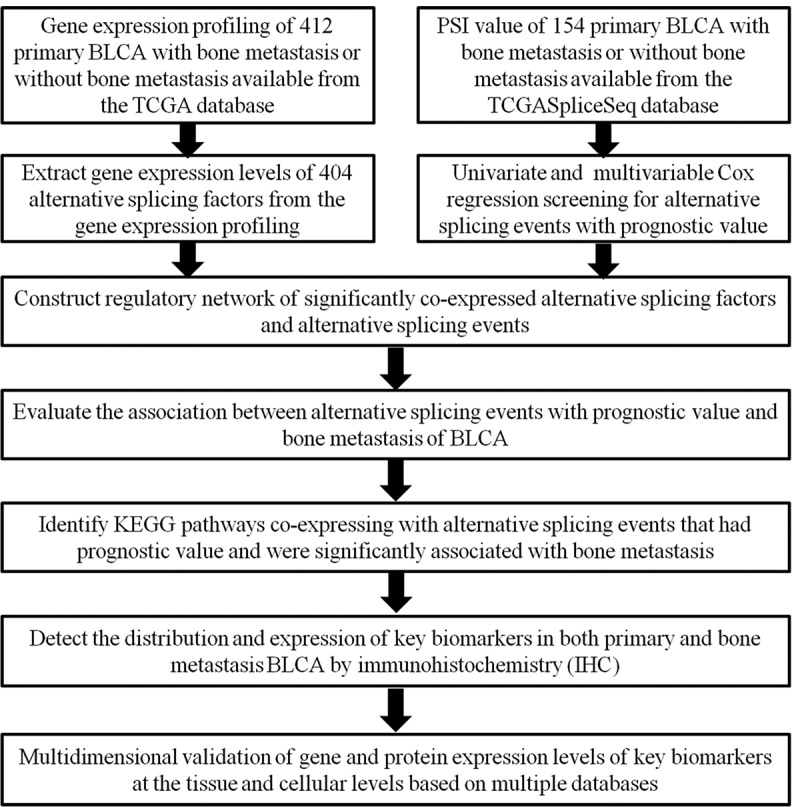
Article overall idea design

**Figure 2.**
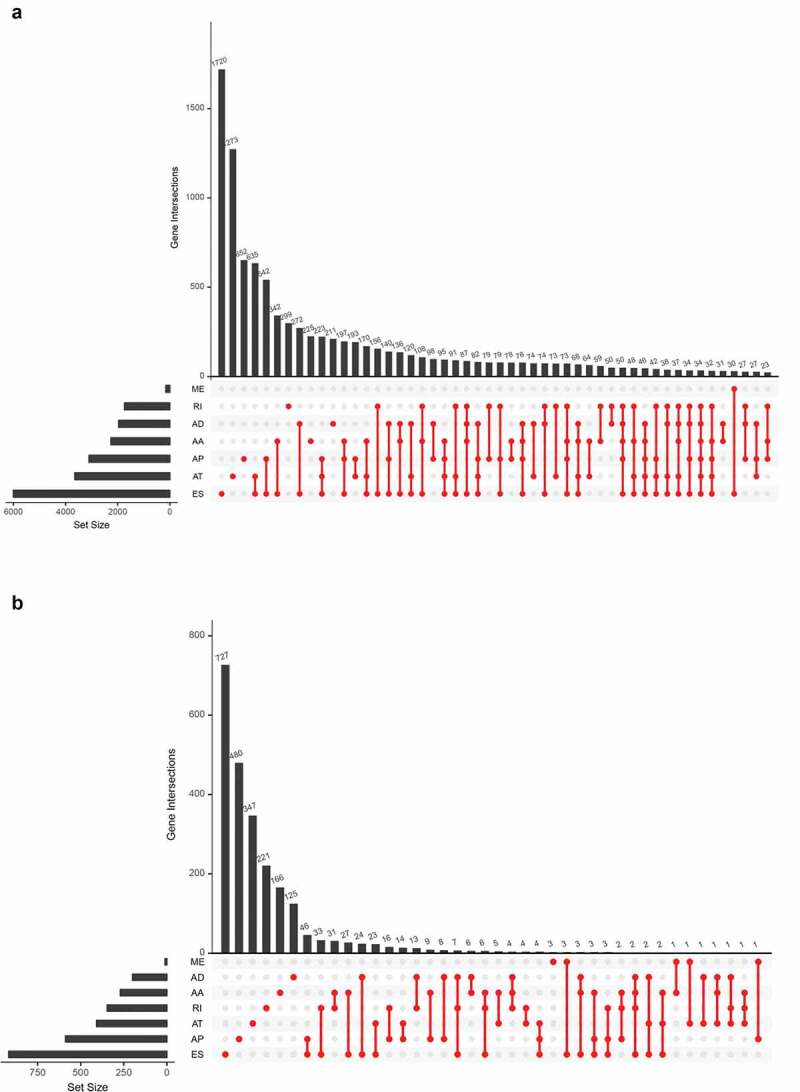
The UpSet plot of SEs and OS-SEs. (a) The number of ASEs in different types of splicing patterns; (b) The number of OS-SEs in different types of splicing patterns

**Abbreviation**: AA, alternate acceptor; AD, alternate donor; AP, alternate promoter; AT, alternate terminator; ES, exon skip; ME, mutually exclusive exons; RI, retained intron.

The overall design of this study is shown in Figure 1. Table S1 summarized the baseline information of 412 patients diagnosed with BLCA. The ASEs profiles and mRNA expression information were downloaded and analyzed. Figure 2a displays the number of genes and ASEs in our data. ASEs of 9,415 genes were recorded, which were classified into 7 patterns: alternate acceptor (AA) events of 2,079 genes, alternate donor (AD) events of 1,814 genes, AP events of 2,924 genes, alternate terminator (AT) events of 3,465 genes, exon skip (ES) events of 5,879 genes, mutually exclusive exons (ME) events of 305 genes and retained intron (RI) events of 1,593 genes.

### Identification of OS-SEs

**Figure 3.**
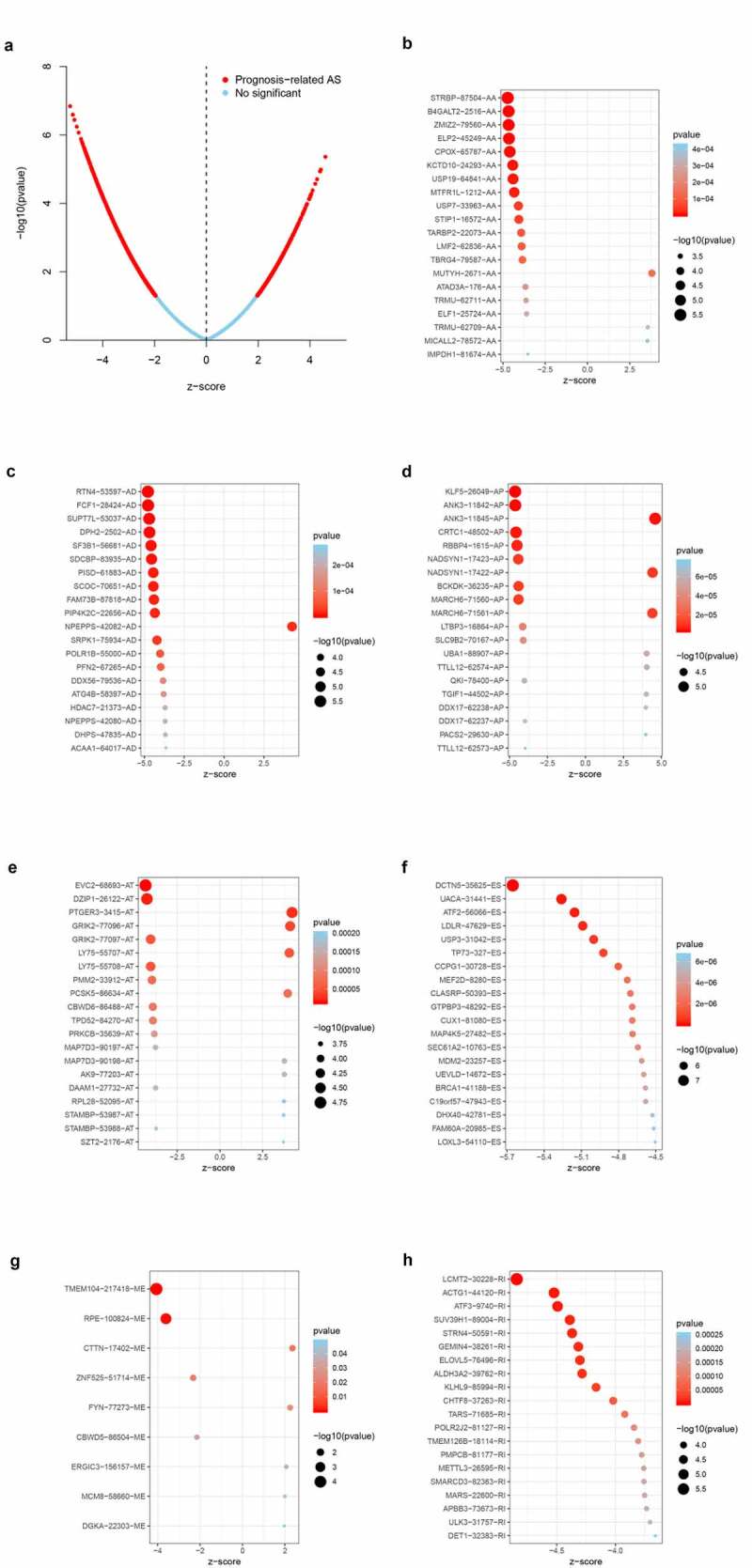
Enrichment analysis of ASEs and bubble charts showing top 20 OS-SEs in seven types of splicing patterns. (a) The volcano plot displaying the prognosis-related and no significant ASEs, respectively. (b) Bubble chart of AA. (c) Bubble chart of AD. (d) Bubble chart of AP. (e) Bubble chart of AT. (f) Bubble chart of ES. (g) Bubble chart of ME. (h) Bubble chart of RI

**Abbreviation**: AA, alternateacceptor; AD, alternate donor; AP, alternate promoter; AT, alternate terminator; ES, exon skip; ME, mutually exclusive exons; RI, retained intron.

The univariate Cox regression analysis was performed to identify the ASEs associated with OS of BLCA patients. Figure 2b shows the OS-SEs of different splicing patterns. The volcano plot showed ASEs that significantly or not significantly associated with BLCA prognosis. As shown in the bubble charts of seven SEs (Figure 3b-Figure 3h), the most relevant OS-SEs for each type were STRBP−87,504− AA (P < 0.001), RTN4 − 53,597− AD (P < 0.001), KLF5 − 26,049− AP (P < 0.001), EVC2 − 68,693− AT (P < 0.001), DCTN5 − 35,625− ES (P < 0.001), TMEM104 − 217,418− ME (P < 0.001) and LCMT2 − 30,228− RI (P < 0.001), respectively.

### Prognostic predictors of OS in BLCA

**Figure 4.**
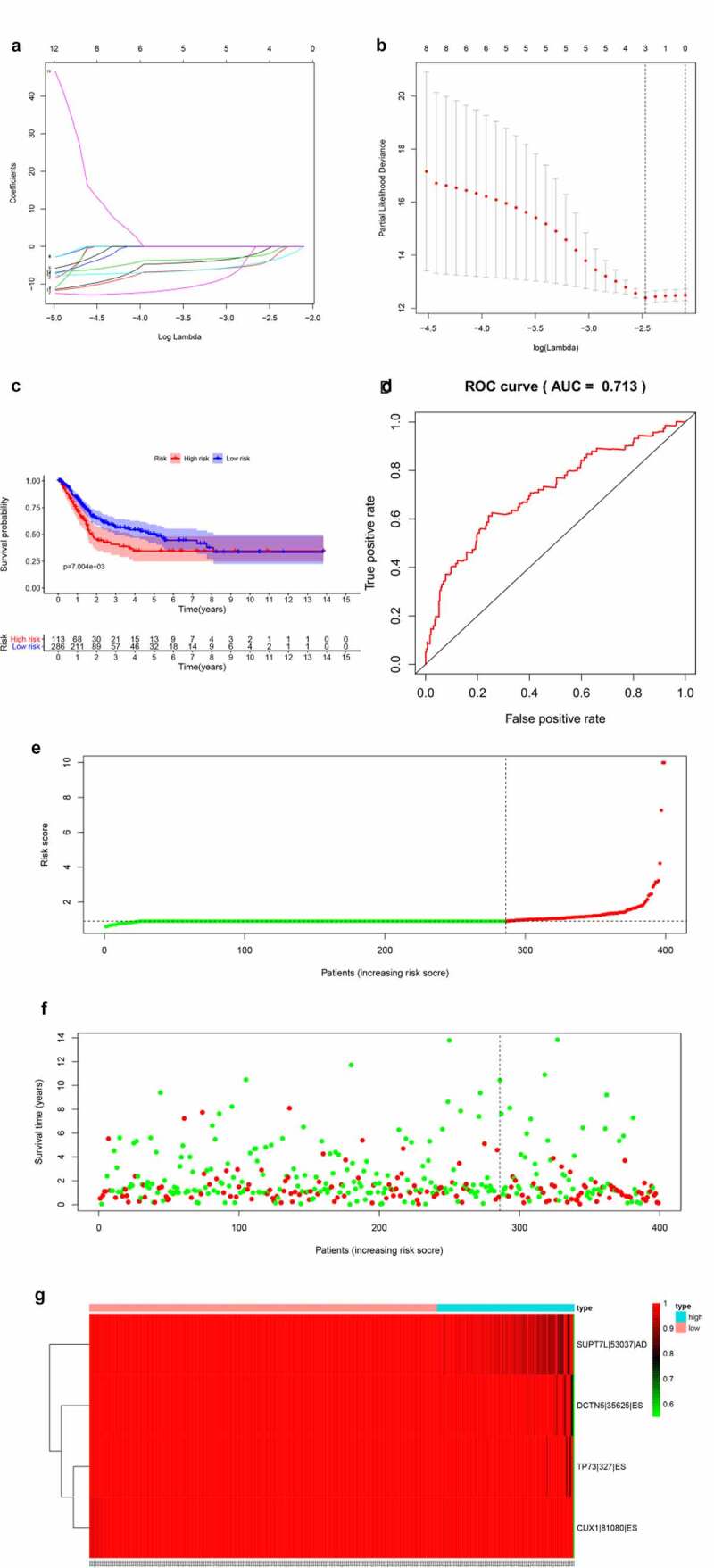
Establishment and assessment of the predict model. (a-b) Lasso regression for OS-SEs screening and removing high correlation genes to prevent over-fitting of the model. (c) Kaplan-Meier survival curves for patients in the low and high subgroups of the predict model demonstrating that risk score could significantly forecast the prognosis of patients with BLCA. (d) ROC curves demonstrating the accuracy of the model (AUC: 0.713). (e) The risk curve of each sample ranking by risk from low to high. (f) The scatter plot showing the trend of change in risk value and the increase in patient mortality as the risk increased and illustrating the clinical status with green and red dots representing survival and death, respectively. (g) The heatmap of expression level of 4 OS-SEs filtered by Lasso regression

**Abbreviation**: AA, alternate acceptor; AD, alternate donor; AP, alternate promoter; AT, alternate terminator; ES, exon skip; ME, mutually exclusive exons; RI, retained intron.

Top 20 OS-SEs in each splicing pattern were selected for the lasso regression model to find valuable prognostic characters for model construction (Figure 4(a,Figure 4b)). Then, multivariate Cox regression analysis was used to construct the model and risk scores of patients were calculated based on our model. The risk curve showed that patients with higher risk scores (red dots) had a poorer prognosis compared with patients with lower risk scores (green dots) (Figure 4(e,Figure 4f)). The K-M survival analysis validated the prognostic value of the risk score of our model (P = 0.007) (Figure 4c). The receiver operating characteristic curve (ROC) also displayed a good accuracy of the constructed predict model (area under the ROC curve, AUC:0.713) (Figure 4d). Figure 4g reveals that SUPT7L-53,037-AD, DCTN5-35,625-ES, TP73-327-ES, CUX1-81,080-ES were less common in patients with high risks.

**Figure 5.**
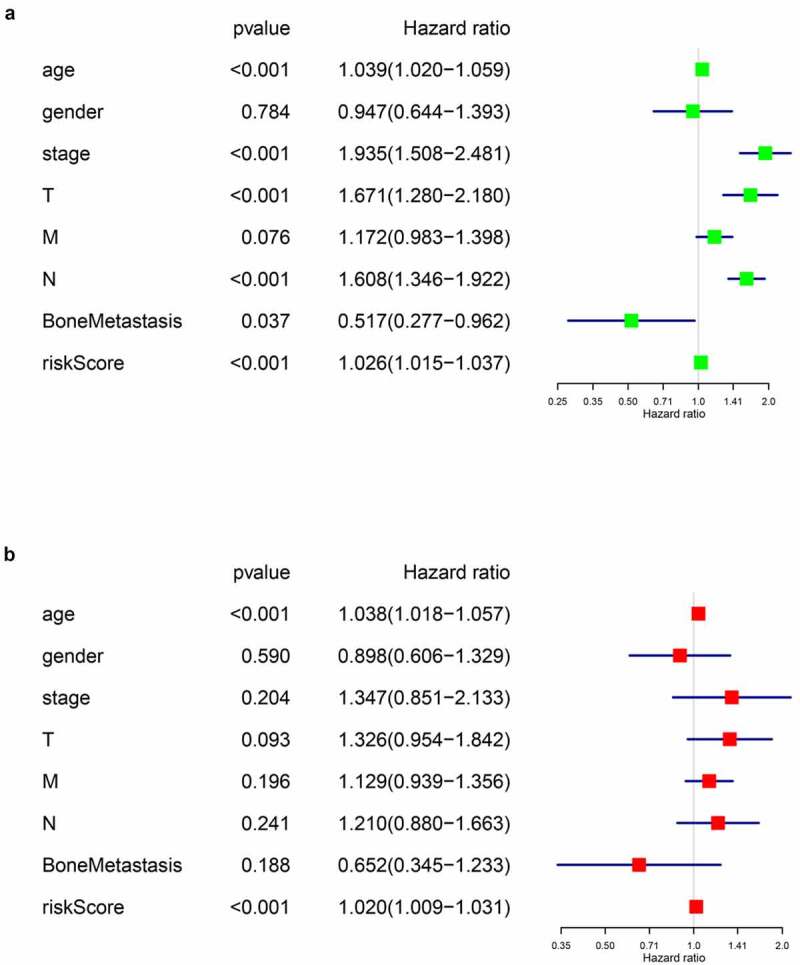
Cox regression analysis for evaluating the independent prognostic value of the risk score. (a) univariate and (b) multivariate Cox regression analysis verify that risk score can be the independent prognostic factor of BLCA

Next, univariate and multivariate Cox regression analysis were performed to evaluate whether the riskscore of our prognostic model was an independent prognostic factor of BLCA patients. Firstly, we assessed the predictive values of the riskscore and baseline information using univariate Cox regression analysis (Figure 5a). Then, characters with statistically significance were imported into the multivariate analysis, and the integrated screening process was depicted in Figure 5(a,Figure 5b). Our results showed that the riskscore of our model was a risk factor of BLCA prognosis independent of age, tumor stage, T or N tumor metastasis status and bone metastasis.

### Correlation between OS-SEs and SF expression

**Figure 6.**
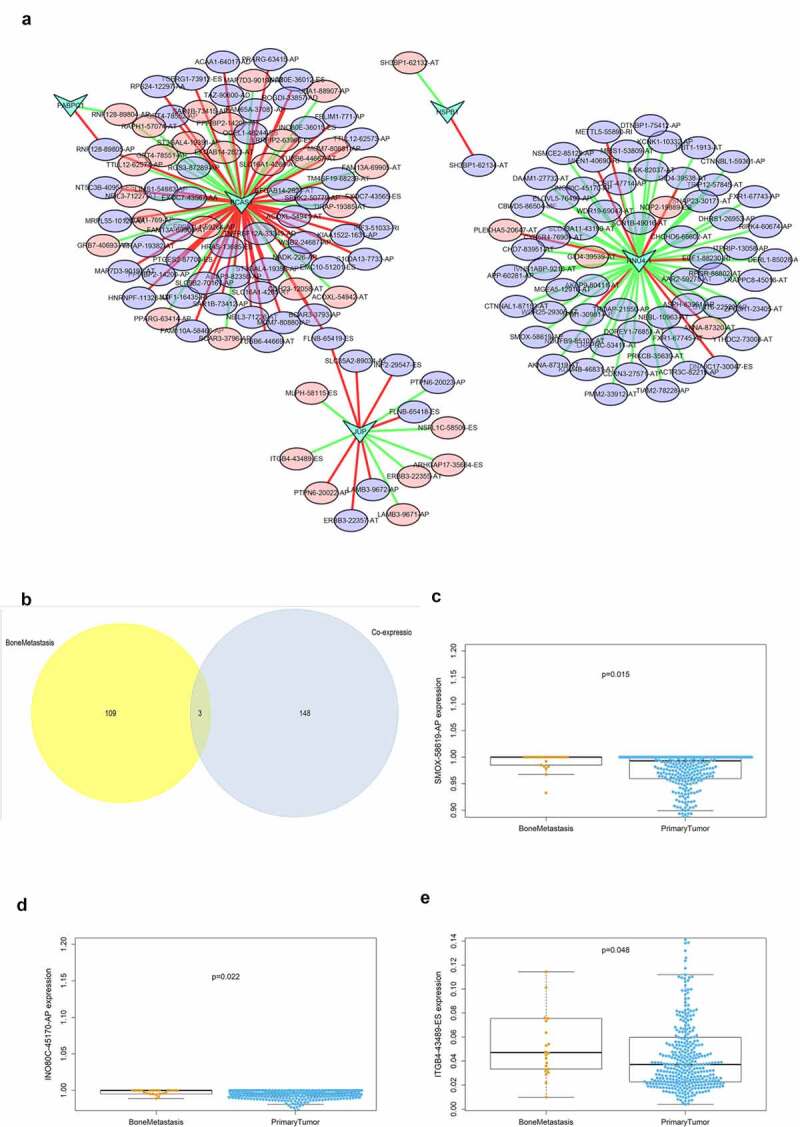
Alternative splicing network and clinical relevance. (a) Regulatory network of significantly co-expressed alternative splicing factors and alternative splicing events. The shape of arrow represents the splicing factor, the red circle shows high risk alternative splicing and the purple circle shows low risk alternative splicing. The red and green lines represent the positive and negative regulatory relationships between AS and SF respectively. (b) Venn plot OS-SEs related to clinical status and bone metastasis. (c) Beeswarm plots displaying SMOX-58,619-AP significantly related to bone metastasis. (d) Beeswarm plots displaying INO80C-45,170-AP significantly related to bone metastasis. (e) Beeswarm plots displaying ITGB4-43,489-ES significantly related to bone metastasis

Figure 6a displays the regulatory network of co-expressed SFs and ASEs. Five key SFs were selected and were significantly correlated with plenty ASEs. In the network, purple ellipses represent adverse OS-SEs and the red ellipses represent favorable OS-SEs. The red lines represent positively association while green lines represent negatively regulation. Then, the ASEs both in the regulatory network and related to bone metastasis were considered as the intersections (Figure 6b). SMOX-58,619-AP (Figure 6c, p = 0.015), INO80C-45,170-AP (Figure 6d, p = 0.022) and ITGB4-43,489-ES (Figure 6e, p = 0.048) were found to be significantly related to both bone metastasis and OS in the Venn plot.

### Co-expression analysis

**Figure 7.**
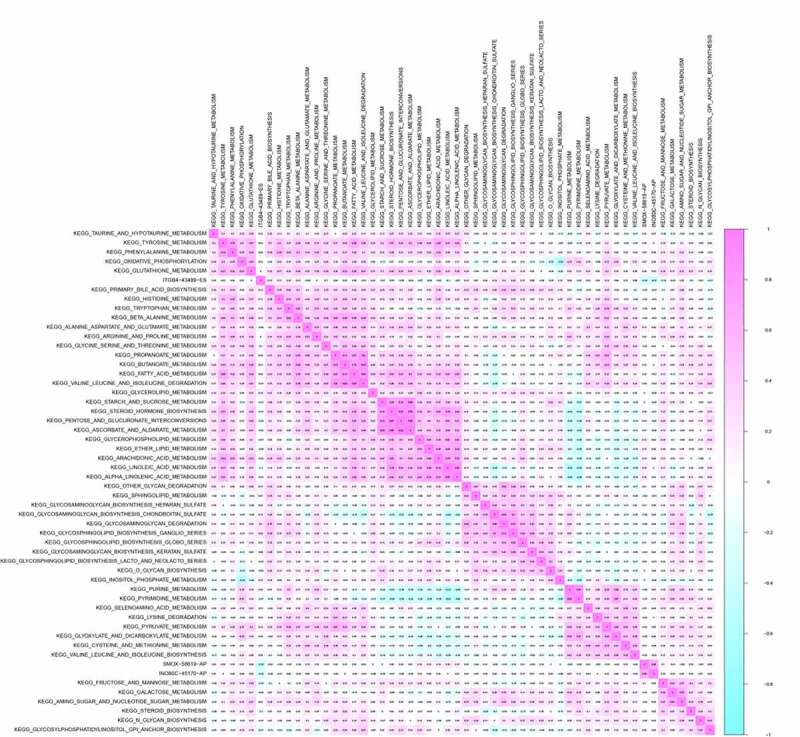
CorHeatmap of KEGG pathways and alternative splicing events that had prognostic value and were significantly associated with bone metastasis. GSVA pathway analysis and univariate Cox regression analysis identified survival related KEGG pathways, and co-expressed alternative splicing events related to prognosis and bone metastasis with survival related KEGG pathways

**Figure 8.**
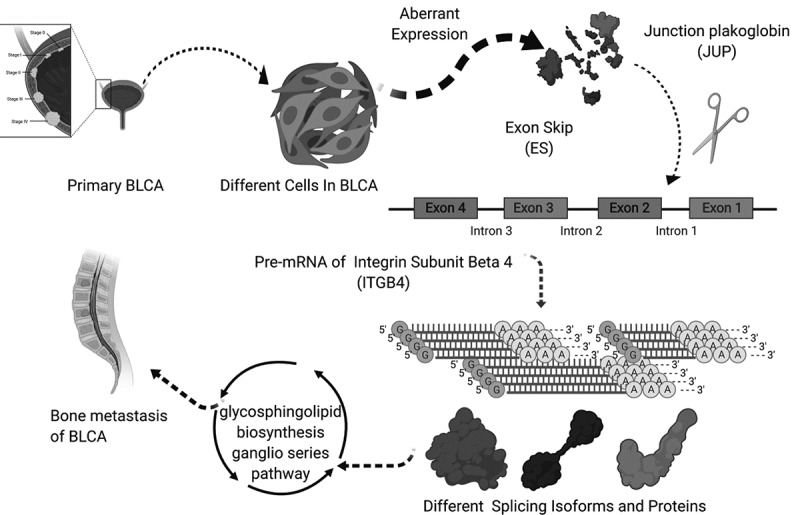
The speculative mechanism diagram including JUP, ITGB4 − 43,489− ES and glycosphingolipid biosynthesis ganglio series pathway

Co-expression analysis was performed on SFs, OS-related ASEs, bone metastasis related ASEs and OS-related KEGG pathways. As shown in Figure 7, ITGB4 − 43,489− ES was positively correlated with the pathways of primary bile acid biosynthesis (R = 0.170), tryptophan metabolism (R = 0.160), glycerolipid metabolism (R = 0.16) and glycosphingolipid biosynthesis ganglio series (R = 0.220) but negatively correlated with the pathway of fructose and mannose metabolism (R = −0.160) and glycosylphosphatidylinositol gpi anchor biosynthesis (R = −0.170). SMOX−58,619− AP was co-expressed with Linoleic acid metabolism (R = 0.180) and Alpha linolenic acid metabolism (R = 0.160). Besides, INO80C−45,170− AP was up-regulated in pyrimidine metabolism. Through multidimensional validation, we speculated that JUP regulating the ITGB4 − 43,489− ES might play a key role in bone metastasis and prognosis of bladder cancer through the glycosphingolipid biosynthesis ganglio series pathway (Figure 8).

### Multidimensional validation based on external databases

In order to detect gene and protein expression levels of key biomarkers of the 7 OS-SEs, we conducted multidimensional validation using multiple databases. Firstly, in pathway unification database, ST8SIA5, ST8SIA1, ST3GAL2, ST3GAL5 and B4GALNT1 were key molecules in glycosphingolipid biosynthesis ganglio series pathway. Then, in UALCAN database, we found JUP and ITGB4 were higher expressed in tumor while ST3GAL5 and ST8SIA1 were lower expressed in tumor (Figure S1A-D). In the database of LinkedOmics, ST8SIA5 was related to OS and tumor stage (Figure S1E, F). In the SurvExpress, ST8SIA5, ST3GAL5 and B4GALNT1 were associated with OS significantly (Figure S1G). In the CCLE database, expression of JUP, ITGB4, ST8SIA5, ST8SIA1, ST3GAL2, ST3GAL5 and B4GALNT1 in pan-cancer were explored (Figure S2A-G). Meanwhile, the GEPIA database showed that JUP, ITGB4, ST3GAL2, ST3GAL5 and B4GALNT1 were higher expressed in tumor samples than in paired normal tissues (Figure S3). Moreover, correlation between JUP and ITGB4 was detected using GEPIA (Figure S7A) and LinkedOmics databases (Figure S7B). Expression of JUP in normal urothelial tissue (Figure S7C) and urothelial carcinoma (Figure S7D), ITGB4 in normal urothelial tissue (Figure S7E) and urothelial carcinoma (Figure S7F) were also shown. Table S2 summarized the results of external validation of BCAS1, ITGB4, ST8SIA5, ST8SIA1, ST3GAL2, ST3GAL5 and B4GALNT1. Finally, Figure 8 depicts the process of our hypothesis: exon skip of ITGB4 was regulated by the SF JUP, which may play an important role in BLCA bone metastasis through the glycosphingolipid biosynthesis ganglio series pathway.

### Cross-validation

To verify the credibility of our model, we divided our dataset into testing and training dataset by a ratio of 3:7, with the proportion of dead patients balanced using lasso regression model (Figure S4 A, B). Baseline information of training, testing and total dataset were severally shown in Table S3, Table S4 and Table S5. Features of models were identified by lasso regression and then were brought into the multivariate Cox regression to calculate the risk score. Patients of training, testing and total dataset were sectionalized into high or low-risk score groups according to the median risk score of specific datasets. Differential expression analysis (Figure S4 C) and K-M survival analysis (Figure S4 D) were performed between high and low-risk groups. The ROCs of three datasets were generated, and the AUCs showed good prognostic value of our models (AUC: training dataset = 0.724; testing dataset = 0.656; total dataset = 0.704). Figure S4 G showed the risk lines of three datasets. Figure S4 H showed the expression of model features in each dataset. We should add that the features here in the total dataset was slightly different with features in our previous model shown in Figure 4g. That was caused by the unfixed training or testing dataset divided by lasso regression model. Though the gene number of features were different in each model, the high AUC of all models proved the good prognostic values of key ASEs in models. These results further verified the prognostic value of our model and model-construction process.

### Validation based on scRNA-seq data, ATAC-seq data and RNA-seq data from MET500 database

The scRNA-seq data of BLCA were exported and analyzed to validate the expression of JUP and ITGB4. Firstly, all BLCA samples were reduced into 10 cellular clusters (Figure S5 A). Genes significantly expressed in each cluster were shown in Figure S5 B. Heatmap of marker genes in each cluster was also generated (Figure S5 C). Cell cycle of cells in each cluster were described in Figure S5 D. Expression of JUK and ITGB4, together with some cell-type markers were highlighted intuitively or quantificationally (Figure S5 E, F). And the results showed a higher expression of JUP and ITGB4 in cells expressing CD44 or CD24, which were cell markers of cancer stem cells. So, we hypothesized that cancer stem cells maybe essential in the regulatory mechanism of JUP and ITGB4. In addition, differential expression analysis of JUP and ITGB4 was performed based on TCGA and MET500 RNA-seq data (Figure S6 A). The ATAC-seq analysis displayed an active regulatory potential of JUP (Figure S6 B) and ITGB4 (Figure S6 C) on chromatin accessibility in BLCA patients.

## Discussion

Bladder cancer was the most common malignant tumor of the urinary system with the highest morbidity and mortality [30]. It had a high degree of malignancy and often presented invasive development. After surgery, the risk of recurrence and metastasis was more than 45% in 1 year. Bone was a widespread metastatic site of solid tumors. Even with cisplatin chemotherapy, patients of bladder cancer with bone metastasis could survive less than 14–15 months [4]. Alternative splicing was considered as one of important biological processes during tumorigenesis and progression [8], particularly in the invasion and metastasis of tumor cells [9–11]. Alternative splicing events were key biomarkers for cancer diagnosis and treatment, as well as potential targets for drug discovery [31], however, few studies focused on the potential role of alternative splicing events in bone metastasis and prognosis of BLCA.

In this study, we firstly found that alternative splicing was associated with the occurrence and progression of bladder cancer, and had a certain relationship with the prognosis of bladder cancer. Three genes at the minimum cross-validation error point in Lasso regression were incorporated into the final Cox regression model as independent prognostic indicators affecting the prognosis of bladder cancer patients. ROC curve showed good evaluation results for the accuracy of the constructed predict model, and univariate and multivariate Cox regression analysis results proved that the prognostic model could be used as an independent prognostic factor. SMOX-58,619-AP, INO80C-45,170-AP and ITGB4-43,489-ES were significantly related to the bone metastasis, splicing factor and survival. The three splicing events were co-expressed with the OS-related KEGG pathways, and after multiple databases, we speculated and constructed a final regulation model. For bladder cancer patients with bone metastasis, JUP could down-regulating the ITGB4 − 43,489− ES by the pathway of glycosphingolipid biosynthesis ganglio series which was also related to the prognosis.

Junction plakoglobin (JUP, γ‐catenin), a member of the armadillo family of proteins [32], is a homolog of β‐catenina and forms distinct complexes with cadherins and desmosomal cadherins, which is a key part of the extracellular matrix [33]. These catenin proteins mediated intercellular interactions and signal transduction between cells [34]. Since JUP is an adhesive protein, the lack of JUP expression can reduce cell–cell contact and increase its proliferation in the body and cancer cells [32]. In this study, by constructing the network of OS-SEs and prognosis-related SFs, we found JUP was one of the SFs that associated OS-SEs, OS and bone metastasis. Besides, a negative regulatory relationship was existed between JUP and ITGB4. Similar to our results, it was reported that JUP was a crucial SF affecting the metastasis and prognosis of other cancers. In oral squamous cell carcinoma, JUP promoted its proliferation, migration, invasion and was a potential prognostic marker [32]; In breast cancer, loss of JUP would trigger the decreasing contact between cells and the increasing the invasion and spread of breast cancer cells [35]; In addition, Syrigos et al. and Rieger et al. found that bladder cancer patients with an abnormal expression of JUP always had poor survival status, and the restoration of plakoglobin expression in bladder carcinoma cell lines could inhibit cell migration and tumorigenic potential [36,37].

Integrin played a major role in signaling networks that promoted angiogenesis and tumor progression [38]. Genetic experiments suggested that tumor cells might be more dependent on specific integrin than normal cells and might be regulated by integrin signals at different stages of tumor progression [39]. Integrin Beta 4 (ITGB4) was the structural component that maintains the hemidesmosomes (HDs) of the epithelial architecture [40]. It was the laminin receptor in tumor cells and angiogenic endothelial cells [41]. Integrin beta4 was characterized by its 1017-amino acid long domain in the beta4 subunit which paired only with the α6 subunit, and the heterodimeric integrin α6β4 played a role in the invasive and metastatic phenotype of various cancers [39,42,43]. Previous studies showed that ITGB4 was highly expressed in a variety of tumors [40,44]. It participated in the proliferation, invasion and metastasis [45–47], and also associated with poor prognosis of some tumors [48,49]. Leng et al. found that ITGB4 could enhance the tumor growth in hepatocellular carcinoma patients and promote lung metastasis by activation of FAK-AKT pathway [46]. In ovarian cancer, the Hh signaling pathway could induce cell migration and invasion through the activation of FAK, which was mediated by ITGB4 [50]. ITGB4 could also serve as a prognostic marker for breast cancer [49].

The up-regulation of ITGB4 in multiple cancer cells indicated that the redistribution of ITGB4 provided favorable conditions for cell proliferation and invasion [40]. In normal epithelial cells, ITGB4 bound to HDs and promoted the anchoring of epithelial cells to the basal membrane. But in cancer cells, ITGB4 was redistributed from HDs to the anterior edges of cells enriched in the lamellar and filamentous feet, enhancing tumor migration and invasion [51,52]. In tumor tissues, phosphorylation of the cytoplasmic tail of ITGB4 led to its release from the semi-desmosome and its interaction with the growth factor receptor [53]. The phosphorylation of the cytoplasmic tail of ITGB4 released integrin α6β4 from hemidesmosomes, which led to its interaction with growth factor receptors and the induction of growth signaling [53,54]. Phosphoinositide 3-kinase (PI3K) and RhoA small gtpase were activated by integrin alpha 6 beta 4 bound to laminin. In addition, the interaction between integrin alpha 6 beta 4 and growth factor receptors included activation signaling pathways of the epidermal growth factor receptor family, such as PI3K AKT, and MAPK signaling was involved in tumorigenesis and metastasis [55]. Therefore, similar to our hypothesis, ITGB4 was associated with bone metastasis of bladder cancer and could be used as a prognostic marker in bladder cancer.

To explore the regulation between JUP and ITGB4, the glycosphingolipid biosynthesis ganglio series pathway was identified as the co-expression signaling pathway through GSVA pathway analysis. Ganglioside (GS) was one kind of sugar sphingolipids containing sialic acid. It was the main component of animal cell membrane [56] and engaged in intercellular recognition, connection, movement and information transmission [57]. It was also associated with tumor differentiation and malignant transformation [58]. GM3, a single sialic acid containing ganglioside, regulated cell adhesion, growth and movement by altering the level of molecular tissue in the synaptic microzone of sugar genes and the activation of co-localization signaling molecules involved in cancer pathogenesis [59]. Previous studies had proved the significant accumulation of GM3 in non-muscle-invasive bladder cancer but a small quantity in muscle-invasive bladder cancer [56]. Furthermore, increased GM3 expression induced growth suppression of bladder cancer cells by brefeldin A [60].

However, there were inevitably some limitations in our study. First of all, the data used in this study was from the public source. Information on other confounding variables, such as smoking, was not available for analysis. But, given the large populations involved we would have anticipated that any differences in background factors would have been evenly distributed via randomization. Secondly, the samples were all from European, which might lead a selection bias. So, a multiple databases validation was performed to reduce this bias by examining the expression levels of co-expressed genes and key molecules in all the other sources we can found. Though there was a lack of laboratory test of our hypothesis in this study, we designed a comprehensive validation on multiple level, including IHA results from HPA database, scRNA-seq and ATAC-seq validation and differential expression analysis based on TCGA and MET500 databases. Favorable results on multiple levels indicated a promising transformation value of our key features in BLCA study. By now, it was the first report to discover that ASEs were involved in GSVA pathway in bone metastases in bladder cancer patients. ASEs were firstly used in the prediction of prognosis in bladder cancer patients. Therefore, our findings could have a nice guiding role for clinicians to make a reasonable prediction for bone metastases for bladder cancer patients.

## Conclusion

In this study, we speculated that ASEs of ITGB4, regulated by the splicing factor JUP, might play a key role in BLCA bone metastasis and prognosis, through the ‘glycosphingolipid biosynthesis ganglio series’ pathway. Based on the comprehensive bioinformatics analysis, a predict model for forecasting the prognosis of BLCA patients was constructed, and its reliability was demonstrated by its high AUC value. The identified alternative splicing events were significantly correlated with bone metastasis and had certain prognostic value for bladder cancer patients.

## Supplementary Material

Supplemental Material

## Figures and Tables

**Table:** Abbreviation

ASEs: alternative splicing events
BLCA: bladder urothelial carcinoma
TCGA: The Cancer Genome
AtlasOS: overall survival
SE: splicing events
SF: splicing factor
AS: alternative splicing
KEGG: Kyoto Encyclopedia of Genes and Genomes
GO: Gene Ontology
PI: prognostic index
ROC: receiver operating characteristic curve
GSVA: Gene Set Variation Analysis
GEPIA: Gene Expression Profiling Interactive Analysis
AA: alternate acceptor
AD: alternate donor
AP: alternate promoter
AT: alternate terminator
ES: exon skip
ME: mutually exclusive exons
RI: retained intron
AUC: area under the ROC curve
TNM stage: T: tumor, N: regional lymph node, M: metastasis
ORF: open reading frame
HDs: hemidesmosomes
JUP: Junction plakoglobin

## Data Availability

The datasets generated and/or analyzed during the current study are available in the TCGA program (https://portal.gdc.cancer.gov) and GEO database (https://www.ncbi.nlm.nih.gov/geo/query/acc.cgi?acc=GSE164041).
