# Supplementary material for: Identification of prognostic and bone metastatic alternative splicing signatures in bladder cancer
Source: Bioengineered. 2021 Aug 17;12(1):5289–304. doi: 10.1080/21655979.2021.1964252 (PMC8806927; doi:10.1080/21655979.2021.1964252)
Supplement: Supplemental Material [file KBIE_A_1964252_SM6467.zip › suppl/Table S1.docx]

| **Table S1** Baseline information of 412 patients disgnosed with bladder cancer. | |
| --- | --- |
| Variables | Total Patients(N=412) |
| **Age,years** |  |
| Mean±SD | 68.10±10.57 |
| Median(Range) | 69 (34 - 90) |
| **Gender** |  |
| Female | 108 (26.21%) |
| Male | 304 (73.79%) |
| **Grade** |  |
| High Grade | 388 (94.17%) |
| Low Grade | 21 (5.10%) |
| unknow | 3 (0.73%) |
| **Stage** |  |
| Stage I | 2 (0.485%) |
| Stage II | 131 (31.80%) |
| Stage III | 141 (34.22%) |
| Stage IV | 136 (33.01%) |
| unknow | 2 (0.485%) |
| **T** |  |
| T0 | 1 (0.24%) |
| T1 | 3 (0.73%) |
| T2 | 38 (9.22%) |
| T2a | 26 (6.31%) |
| T2b | 56 (13.59%) |
| T3 | 43 (10.44%) |
| T3a | 71 (17.23%) |
| T3b | 82 (19.90%) |
| T4 | 11 (2.67%) |
| T4a | 43 (19.44%) |
| T4b | 5 (1.21%) |
| TX | 1 (0.24%) |
| unknow | 32 (7.77%) |
| **M** |  |
| M0 | 196 (47.57%) |
| M1 | 11 (2.67%) |
| MX | 202 (49.03%) |
| unknow | 3 (0.73%) |
| **N** |  |
| N0 | 239 (58.01%) |
| N1 | 47 (11.41%) |
| N2 | 76 (18.45%) |
| N3 | 8 (1.94%) |
| NX | 36 (8.74%) |
| unknow | 6 (1.46%) |
| **Bone Metastasis** |  |
| Yes | 23 (5.58%) |
| No | 389 (94.42%) |
| **Abbreviations:**SD,Standard deviation; T,tumor; M,metastasis; N,regional lymph node. | |
