# Supplementary material for: Identification of prognostic and bone metastatic alternative splicing signatures in bladder cancer
Source: Bioengineered. 2021 Aug 17;12(1):5289–304. doi: 10.1080/21655979.2021.1964252 (PMC8806927; doi:10.1080/21655979.2021.1964252)
Supplement: Supplemental Material [file KBIE_A_1964252_SM6467.zip › suppl/Table S2.docx]

| Table S2 The external validation of JUP, ITGB4, ST8SIA5, ST8SIA1, ST3GAL2, ST3GAL5 and B4GALNT1. | | | | | | | |  |
| --- | --- | --- | --- | --- | --- | --- | --- | --- |
| **Database** | **JUP** | **ITGB4** | **ST8SIA5** | **ST8SIA1** | **ST3GAL2** | **ST3GAL5** | **B4GALNT1** | |
| **UALCAN** | Expression:P = 0.014 | Expression:P < 0.001 | Expression:P = 0.125 | Expression:P < 0.001 | Expression:P = 0.070 | Expression:P = 0.036 | Expression:P = 0.088 | |
| **LinkedOmics** | K-M analysis:P = 0.200 | K-M analysis:P = 0.386 | K-M analysis:P = 0.014 | K-M analysis:P = 0.820 | K-M analysis:P = 0.765 | K-M analysis:P = 0.464 | K-M analysis:P = 0.444 | |
|  | M:P = 0.968 | M:P = 0.589 | M:P = 0.259 | M:P = 0.898 | M:P = 0.134 | M:P = 0.505 | M:P = 0.883 | |
|  | Stage:P = 0.188 | Stage:P = 0.588 | Stage:P = 0.676 | Stage:P < 0.001 | Stage:P = 0.071 | Stage:P = 0.405 | Stage:P = 0.407 | |
| **CCLE** | upregulated in tumor | upregulated in tumor | downregulated in tumor | downregulated in tumor | upregulated in tumor | upregulated in tumor | upregulated in tumor | |
| **SurvExpress** | K-M analysis:P = 0.633 | K-M analysis:P = 0.760 | K-M analysis:P = 0.044 | K-M analysis:P = 0.112 | K-M analysis:P = 0.061 | K-M analysis:P = 0.013 | K-M analysis:P = 0.006 | |
| **GEPIA** | upregulated in tumor | upregulated in tumor | no significant difference | downregulated in tumor | upregulated in tumor | upregulated in tumor | upregulated in tumor | |
| **The Human Protein Atlas** | Tumor high | Tumor not detected | Tumor not detected | Tumor medium | Tumor medium | Tumor low | Tumor not detected | |
|  | Normal high | Normal medium | Normal not detected | Normal medium | Normal not detected | Normal not detected | Normal not detected | |
