# Supplementary material for: Identification of prognostic and bone metastatic alternative splicing signatures in bladder cancer
Source: Bioengineered. 2021 Aug 17;12(1):5289–304. doi: 10.1080/21655979.2021.1964252 (PMC8806927; doi:10.1080/21655979.2021.1964252)
Supplement: Supplemental Material [file KBIE_A_1964252_SM6467.zip › suppl/Table S3.docx]

**Table S3 The baseline information of training dataset.**

| **Covariates** | **Risk** | **Total** | **high** | **low** | **chi** | **Pvalue** |
| --- | --- | --- | --- | --- | --- | --- |
| **age** | <=65 | 112(40%) | 52(37.14%) | 60(42.86%) | 0.7292 | 0.3932 |
| **age** | >65 | 168(60%) | 88(62.86%) | 80(57.14%) |  |  |
| **gender** | FEMALE | 75(26.79%) | 38(27.14%) | 37(26.43%) | 0 | 1 |
| **gender** | MALE | 205(73.21%) | 102(72.86%) | 103(73.57%) |  |  |
| **grade** | High Grade | 260(92.86%) | 137(97.86%) | 123(87.86%) | 12.176 | 5.00E-04 |
| **grade** | Low Grade | 17(6.07%) | 1(0.71%) | 16(11.43%) |  |  |
| **grade** | unknow | 3(1.07%) | 2(1.43%) | 1(0.71%) |  |  |
| **stage** | Stage I | 2(0.71%) | 0(0%) | 2(1.43%) | 27.0689 | 0 |
| **stage** | Stage II | 84(30%) | 23(16.43%) | 61(43.57%) |  |  |
| **stage** | Stage III | 98(35%) | 59(42.14%) | 39(27.86%) |  |  |
| **stage** | Stage IV | 95(33.93%) | 57(40.71%) | 38(27.14%) |  |  |
| **stage** | unknow | 1(0.36%) | 1(0.71%) | 0(0%) |  |  |
| **T** | T0 | 1(0.36%) | 0(0%) | 1(0.71%) | 23.498 | 3.00E-04 |
| **T** | T1 | 2(0.71%) | 0(0%) | 2(1.43%) |  |  |
| **T** | T2 | 78(27.86%) | 25(17.86%) | 53(37.86%) |  |  |
| **T** | T3 | 135(48.21%) | 82(58.57%) | 53(37.86%) |  |  |
| **T** | T4 | 43(15.36%) | 26(18.57%) | 17(12.14%) |  |  |
| **T** | TX | 21(7.5%) | 7(5%) | 14(10%) |  |  |
| **M** | M0 | 137(48.93%) | 51(36.43%) | 86(61.43%) | 18.0601 | 1.00E-04 |
| **M** | M1 | 8(2.86%) | 6(4.29%) | 2(1.43%) |  |  |
| **M** | MX | 135(48.21%) | 83(59.29%) | 52(37.14%) |  |  |
| **N** | N0 | 164(58.57%) | 73(52.14%) | 91(65%) | 8.2405 | 0.0832 |
| **N** | N1 | 33(11.79%) | 23(16.43%) | 10(7.14%) |  |  |
| **N** | N2 | 51(18.21%) | 29(20.71%) | 22(15.71%) |  |  |
| **N** | N3 | 7(2.5%) | 3(2.14%) | 4(2.86%) |  |  |
| **N** | NX | 25(8.93%) | 12(8.57%) | 13(9.29%) |  |  |
