# Supplementary material for: Identification of prognostic and bone metastatic alternative splicing signatures in bladder cancer
Source: Bioengineered. 2021 Aug 17;12(1):5289–304. doi: 10.1080/21655979.2021.1964252 (PMC8806927; doi:10.1080/21655979.2021.1964252)
Supplement: Supplemental Material [file KBIE_A_1964252_SM6467.zip › suppl/Table S4.docx]

**Table S4 The baseline information of testing dataset.**

| **Covariates** | **Risk** | **Total** | **high** | **low** | **chi** | **Pvalue** |
| --- | --- | --- | --- | --- | --- | --- |
| **age** | <=65 | 47(39.5%) | 15(27.27%) | 32(50%) | 5.478 | 0.0193 |
| **age** | >65 | 72(60.5%) | 40(72.73%) | 32(50%) |  |  |
| **gender** | FEMALE | 28(23.53%) | 12(21.82%) | 16(25%) | 0.0366 | 0.8483 |
| **gender** | MALE | 91(76.47%) | 43(78.18%) | 48(75%) |  |  |
| **grade** | High Grade | 116(97.48%) | 55(100%) | 61(95.31%) | 1.0813 | 0.2984 |
| **grade** | Low Grade | 3(2.52%) | 0(0%) | 3(4.69%) |  |  |
| **stage** | Stage II | 43(36.13%) | 16(29.09%) | 27(42.19%) | 6.3349 | 0.0421 |
| **stage** | Stage III | 39(32.77%) | 16(29.09%) | 23(35.94%) |  |  |
| **stage** | Stage IV | 36(30.25%) | 23(41.82%) | 13(20.31%) |  |  |
| **stage** | unknow | 1(0.84%) | 0(0%) | 1(1.56%) |  |  |
| **T** | T1 | 1(0.84%) | 0(0%) | 1(1.56%) | 5.2491 | 0.2627 |
| **T** | T2 | 38(31.93%) | 16(29.09%) | 22(34.38%) |  |  |
| **T** | T3 | 54(45.38%) | 30(54.55%) | 24(37.5%) |  |  |
| **T** | T4 | 14(11.76%) | 6(10.91%) | 8(12.5%) |  |  |
| **T** | TX | 12(10.08%) | 3(5.45%) | 9(14.06%) |  |  |
| **M** | M0 | 57(47.9%) | 22(40%) | 35(54.69%) | 2.7861 | 0.2483 |
| **M** | M1 | 3(2.52%) | 2(3.64%) | 1(1.56%) |  |  |
| **M** | MX | 59(49.58%) | 31(56.36%) | 28(43.75%) |  |  |
| **N** | N0 | 68(57.14%) | 26(47.27%) | 42(65.62%) | 8.3896 | 0.0386 |
| **N** | N1 | 11(9.24%) | 6(10.91%) | 5(7.81%) |  |  |
| **N** | N2 | 24(20.17%) | 17(30.91%) | 7(10.94%) |  |  |
| **N** | NX | 16(13.45%) | 6(10.91%) | 10(15.62%) |  |  |
