# Supplementary material for: Identification of prognostic and bone metastatic alternative splicing signatures in bladder cancer
Source: Bioengineered. 2021 Aug 17;12(1):5289–304. doi: 10.1080/21655979.2021.1964252 (PMC8806927; doi:10.1080/21655979.2021.1964252)
Supplement: Supplemental Material [file KBIE_A_1964252_SM6467.zip › suppl/Table S5.docx]

**Table S5 The baseline information of total dataset.**

| **Covariates** | **Risk** | **Total** | **high** | **low** | **chi** | **Pvalue** |
| --- | --- | --- | --- | --- | --- | --- |
| **age** | <=65 | 159(39.85%) | 67(34.36%) | 92(45.1%) | 4.3594 | 0.0368 |
| **age** | >65 | 240(60.15%) | 128(65.64%) | 112(54.9%) |  |  |
| **gender** | FEMALE | 103(25.81%) | 50(25.64%) | 53(25.98%) | 0 | 1 |
| **gender** | MALE | 296(74.19%) | 145(74.36%) | 151(74.02%) |  |  |
| **grade** | High Grade | 376(94.24%) | 192(98.46%) | 184(90.2%) | 14.3369 | 2.00E-04 |
| **grade** | Low Grade | 20(5.01%) | 1(0.51%) | 19(9.31%) |  |  |
| **grade** | unknow | 3(0.75%) | 2(1.03%) | 1(0.49%) |  |  |
| **stage** | Stage I | 2(0.5%) | 0(0%) | 2(0.98%) | 28.3695 | 0 |
| **stage** | Stage II | 127(31.83%) | 39(20%) | 88(43.14%) |  |  |
| **stage** | Stage III | 137(34.34%) | 75(38.46%) | 62(30.39%) |  |  |
| **stage** | Stage IV | 131(32.83%) | 80(41.03%) | 51(25%) |  |  |
| **stage** | unknow | 2(0.5%) | 1(0.51%) | 1(0.49%) |  |  |
| **T** | T0 | 1(0.25%) | 0(0%) | 1(0.49%) | 26.2382 | 1.00E-04 |
| **T** | T1 | 3(0.75%) | 0(0%) | 3(1.47%) |  |  |
| **T** | T2 | 116(29.07%) | 41(21.03%) | 75(36.76%) |  |  |
| **T** | T3 | 189(47.37%) | 112(57.44%) | 77(37.75%) |  |  |
| **T** | T4 | 57(14.29%) | 32(16.41%) | 25(12.25%) |  |  |
| **T** | TX | 33(8.27%) | 10(5.13%) | 23(11.27%) |  |  |
| **M** | M0 | 194(48.62%) | 73(37.44%) | 121(59.31%) | 19.9149 | 0 |
| **M** | M1 | 11(2.76%) | 8(4.1%) | 3(1.47%) |  |  |
| **M** | MX | 194(48.62%) | 114(58.46%) | 80(39.22%) |  |  |
| **N** | N0 | 232(58.15%) | 99(50.77%) | 133(65.2%) | 13.8473 | 0.0078 |
| **N** | N1 | 44(11.03%) | 29(14.87%) | 15(7.35%) |  |  |
| **N** | N2 | 75(18.8%) | 46(23.59%) | 29(14.22%) |  |  |
| **N** | N3 | 7(1.75%) | 3(1.54%) | 4(1.96%) |  |  |
| **N** | NX | 41(10.28%) | 18(9.23%) | 23(11.27%) |  |  |
