# Supplementary figures and images for: Identification of prognostic and bone metastatic alternative splicing signatures in bladder cancer
Source: Bioengineered. 2021 Aug 17;12(1):5289–304. doi: 10.1080/21655979.2021.1964252 (PMC8806927; doi:10.1080/21655979.2021.1964252)

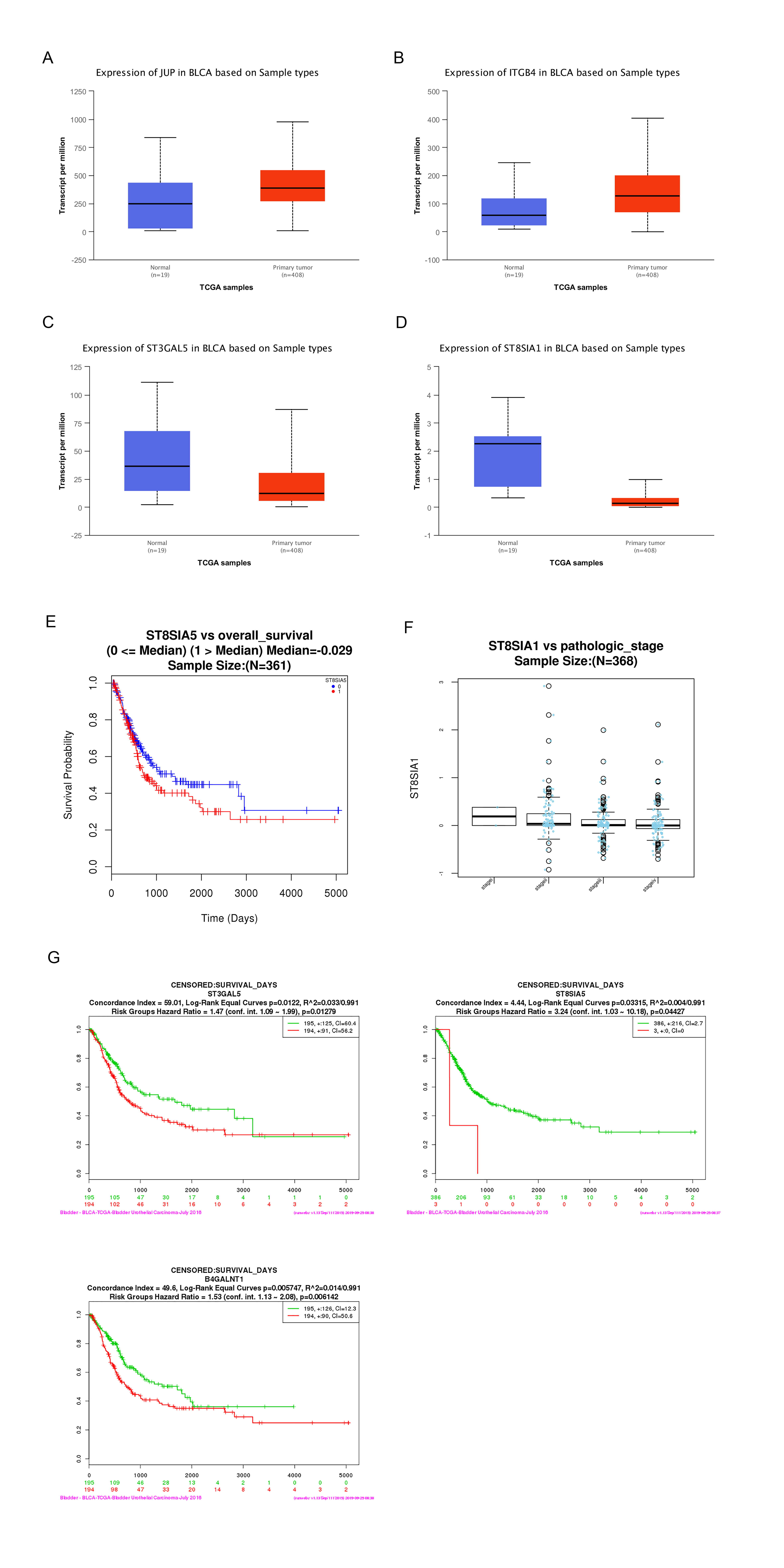

Supplement: Supplemental Material [file KBIE_A_1964252_SM6467.zip › suppl/Fig S1.jpg]

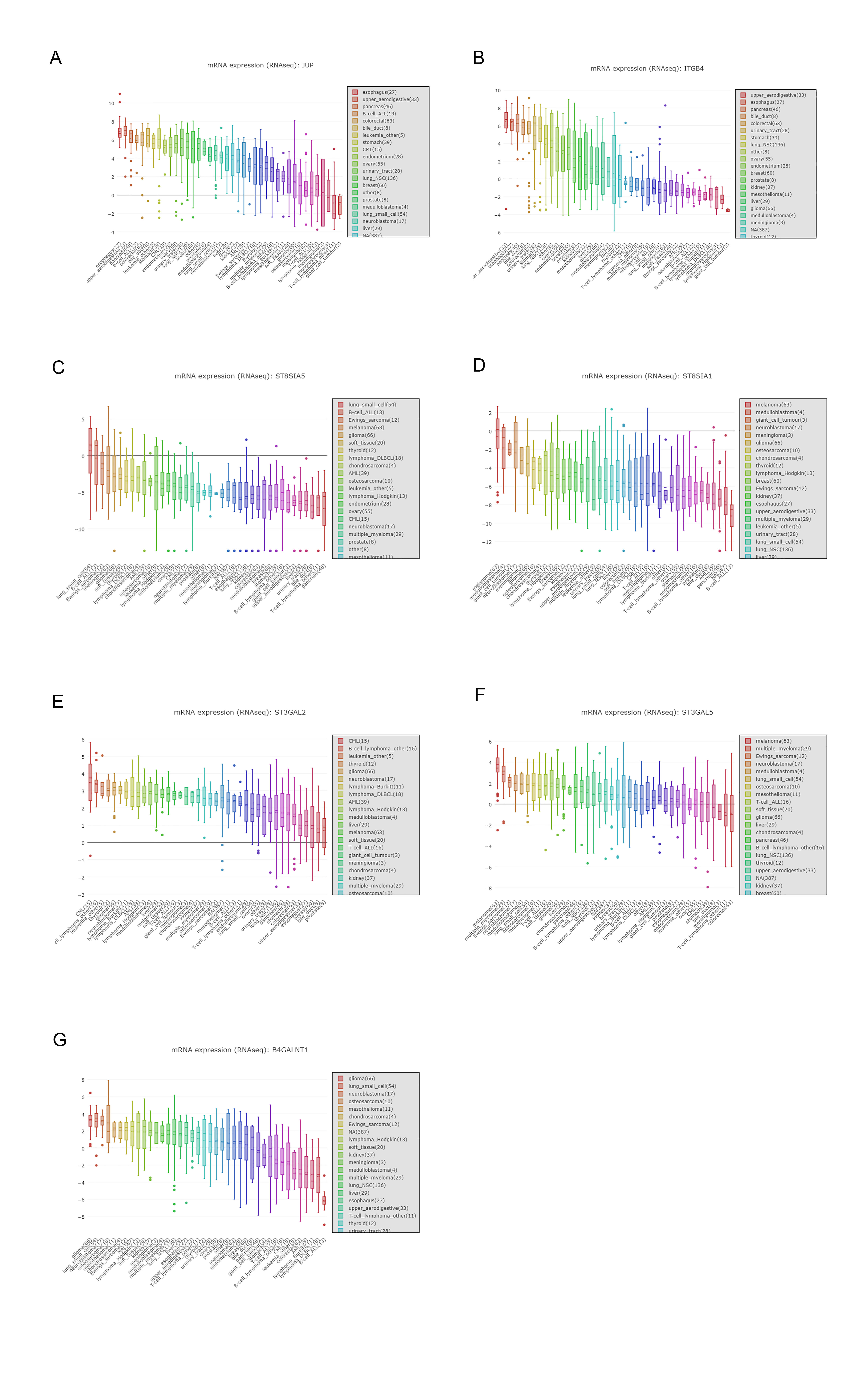

Supplement: Supplemental Material [file KBIE_A_1964252_SM6467.zip › suppl/Fig S2.jpg]

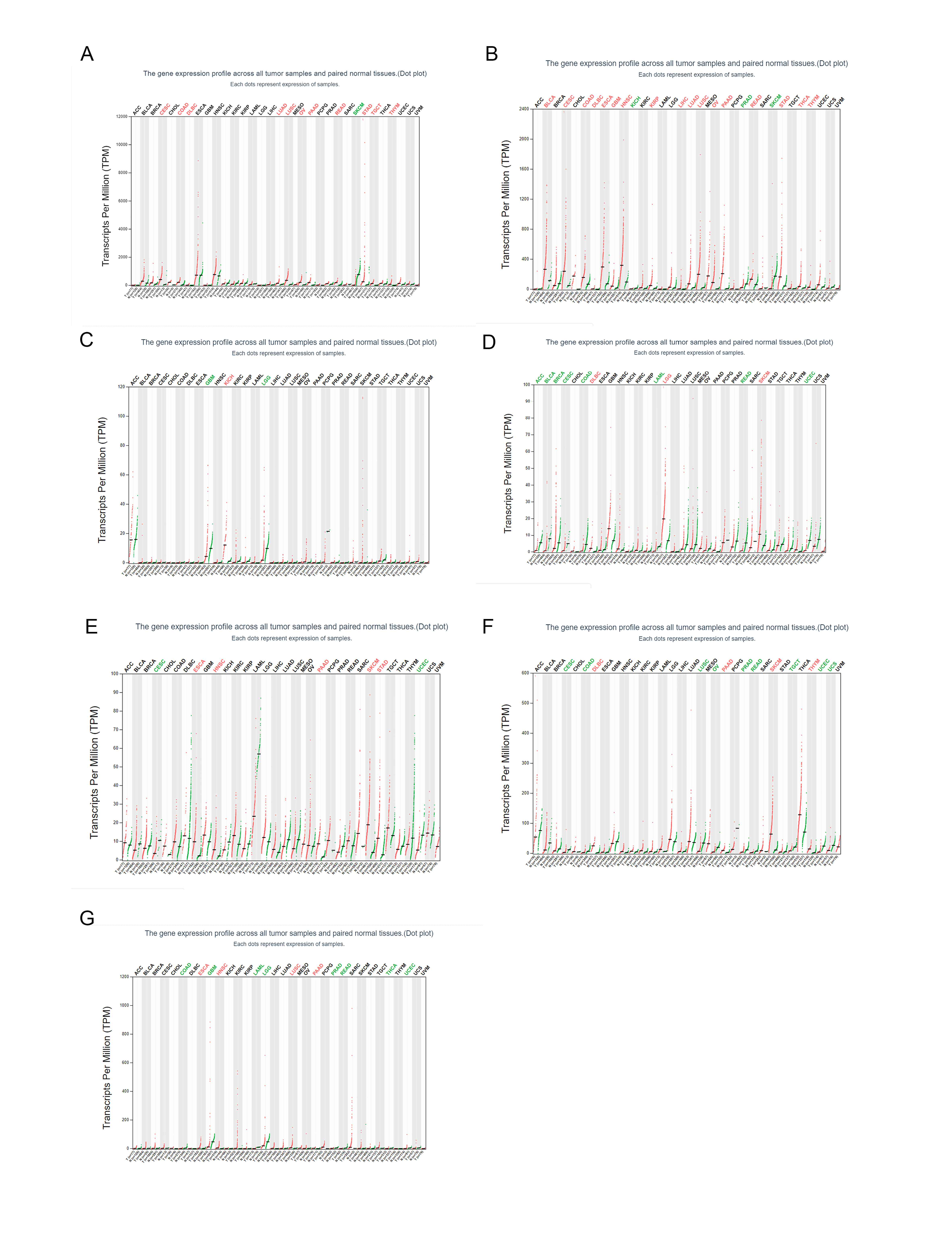

Supplement: Supplemental Material [file KBIE_A_1964252_SM6467.zip › suppl/Fig S3.jpg]

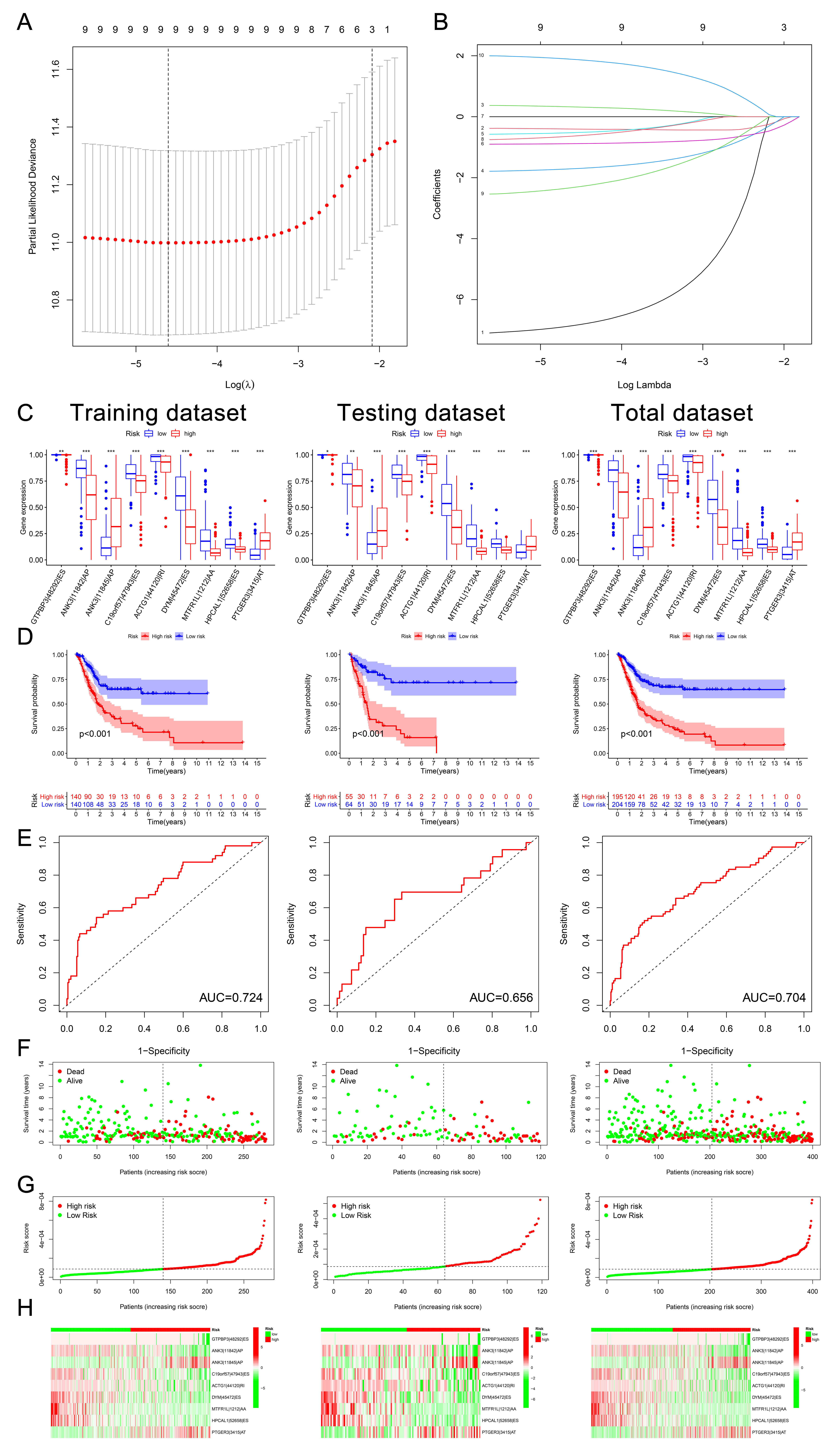

Supplement: Supplemental Material [file KBIE_A_1964252_SM6467.zip › suppl/Fig S4.jpg]

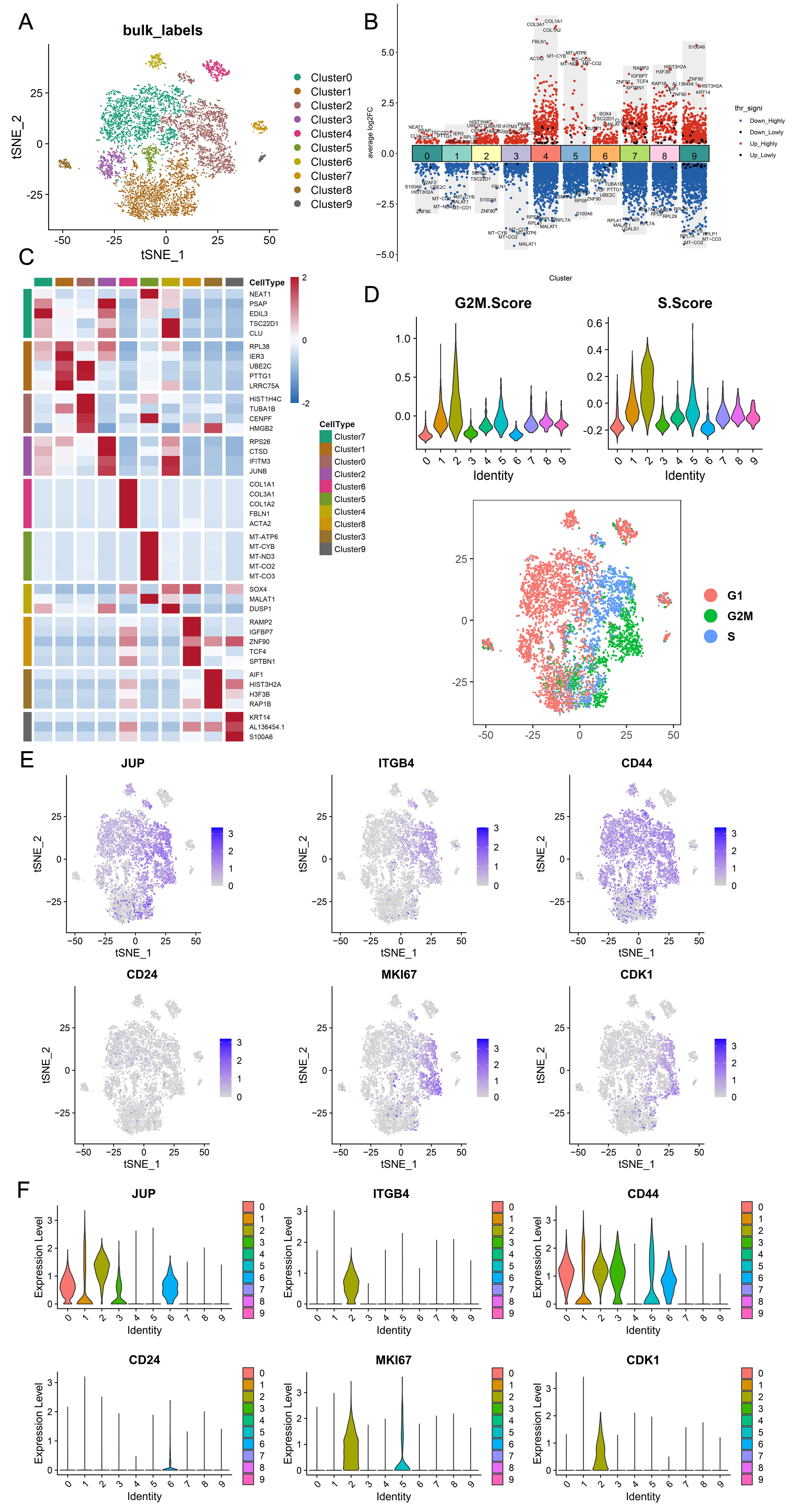

Supplement: Supplemental Material [file KBIE_A_1964252_SM6467.zip › suppl/Fig S5.jpg]

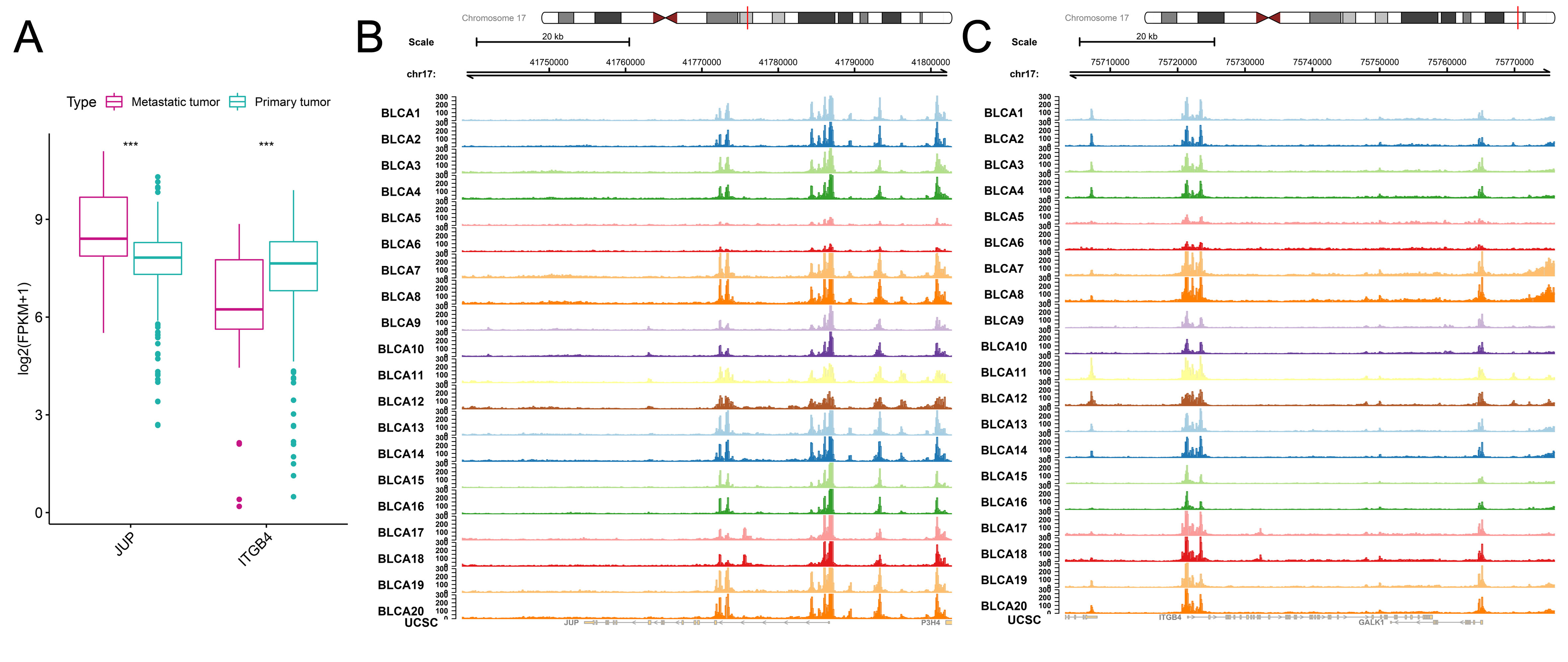

Supplement: Supplemental Material [file KBIE_A_1964252_SM6467.zip › suppl/Fig S6.jpg]

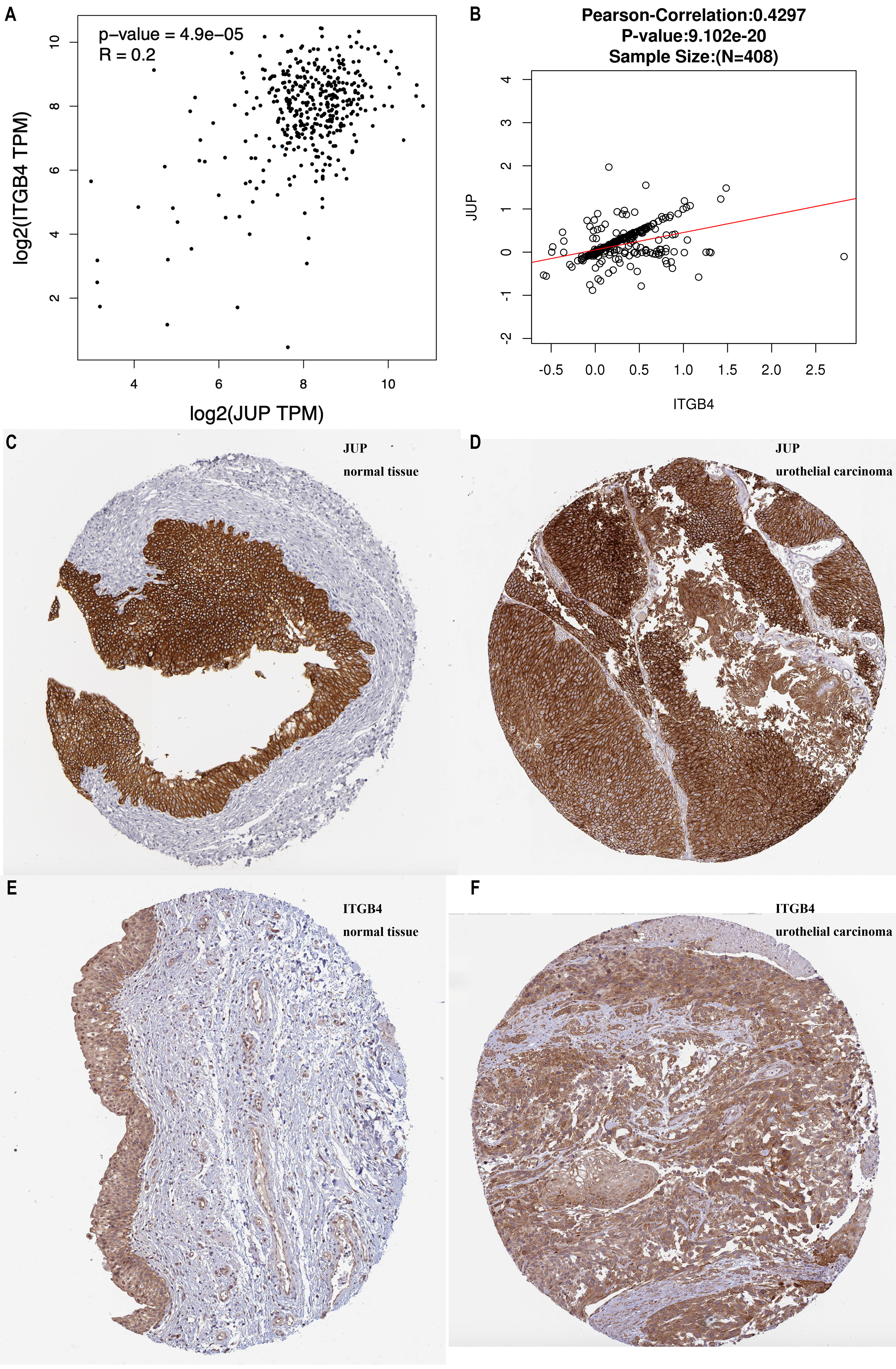

Supplement: Supplemental Material [file KBIE_A_1964252_SM6467.zip › suppl/Fig S7.jpg]
